# Information encoded in a network of inflammation proteins predicts clinical outcome after myocardial infarction

**DOI:** 10.1186/1755-8794-4-59

**Published:** 2011-07-14

**Authors:** Francisco J Azuaje, Sophie Rodius, Lu Zhang, Yvan Devaux, Daniel R Wagner

**Affiliations:** 1 Laboratory of Cardiovascular Research, Public Research Centre for Health (CRP-Santé), L-1150, Luxembourg; 2 Division of Cardiology, Centre Hospitalier, L-1210, Luxembourg

## Abstract

**Background:**

Inflammation plays an important role in cardiac repair after myocardial infarction (MI). Nevertheless, the systems-level characterization of inflammation proteins in MI remains incomplete. There is a need to demonstrate the potential value of molecular network-based approaches to translational research. We investigated the interplay of inflammation proteins and assessed network-derived knowledge to support clinical decisions after MI. The main focus is the prediction of clinical outcome after MI.

**Methods:**

We assembled My-Inflamome, a network of protein interactions related to inflammation and prognosis in MI. We established associations between network properties, disease biology and capacity to distinguish between prognostic categories. The latter was tested with classification models built on blood-derived microarray data from post-MI patients with different outcomes. This was followed by experimental verification of significant associations.

**Results:**

My-Inflamome is organized into modules highly specialized in different biological processes relevant to heart repair. Highly connected proteins also tend to be high-traffic components. Such bottlenecks together with genes extracted from the modules provided the basis for novel prognostic models, which could not have been uncovered by standard analyses. Modules with significant involvement in transcriptional regulation are targeted by a small set of microRNAs. We suggest a new panel of gene expression biomarkers (TRAF2, SHKBP1 and UBC) with high discriminatory capability. Follow-up validations reported promising outcomes and motivate future research.

**Conclusion:**

This study enhances understanding of the interaction network that executes inflammatory responses in human MI. Network-encoded information can be translated into knowledge with potential prognostic application. Independent evaluations are required to further estimate the clinical relevance of the new prognostic genes.

## Background

Cardiovascular disease is a major cause of death worldwide. Myocardial infarction (MI) often leads to heart failure (HF), which makes MI a leading source of morbidity and hospitalizations [1]. Different inflammation biomarkers with diagnostic and prognostic applications have been deployed in the clinical setting [2,3]. Post-MI cell death triggers the activation of several complex inflammatory processes to clear dead cells and initiate heart tissue regeneration [4-7]. It has been suggested that such a coordinated regulation is fundamental to enable cardiac repair and recovery after MI [8]. C-reactive protein (CRP) and other pro-inflammatory cytokines are widely-applied biomarkers in the routine clinical setting [2,6,9]. For example, CRP's capacity to identify patients with high risk of developing HF has been demonstrated in different studies, including the Framingham Heart Study [10], though this can be achieved at the expense of low specificity [2].

Notwithstanding the widely-accepted application of different prognostic biomarkers after MI, including indicators of inflammation, there is a need to provide new insights into the complex mechanisms leading to HF. In addition, currently there is no single biomarker capable to accurately classify patients on the basis of their post-MI outcome [2]. Standard research approaches have been based on the analysis of differential expression of putative biomarkers, using gene or protein expression values [3,4,11]. The need to provide mechanistic understanding at a systems level, while improving the predictive ability of candidate biomarkers, makes network-based approaches a pertinent new research direction in this clinical area. This strategy not only entails the representation and analysis of networks of putative biomarkers, but also benefits from the integration of multiple sources of omic information.

The application of systems-driven approaches to discovering prognostic biomarkers and understanding disease biology has received relatively more attention in the areas of cancer and neurodegenerative diseases [12-14]. We have previously demonstrated its potential to guide new knowledge discovery in translational research of MI [15-19]. For instance, we have established quantitative connections between network topological features, modularity and prognostic outcome in post-MI [16]. Moreover, we have explored the prognostic value of new biomarkers relevant to angiogenesis and cardiac repair [15,18]. Based on these outcomes and the lack of systems biology research of inflammation in the specific context of MI, we set out to investigate a comprehensive set of inflammation-implicated protein interactions and its potential prognostic value post-MI. The main focus of this investigation was to predict clinical outcome after MI, in particular left ventricular dysfunction.

### Objective

The objective of our investigation is three-fold:

1. To characterize a comprehensive compendium of protein interactions relevant to inflammation and (post-MI) HF, through the implementation of an integrative biological network approach.

2. To establish qualitative and quantitative connections between network properties, disease biology and prognostic outcomes.

3. To test the potential of new network-derived prognostic biomarkers of post-MI ventricular dysfunction.

## Methods

### Research framework

**Figure 1:**
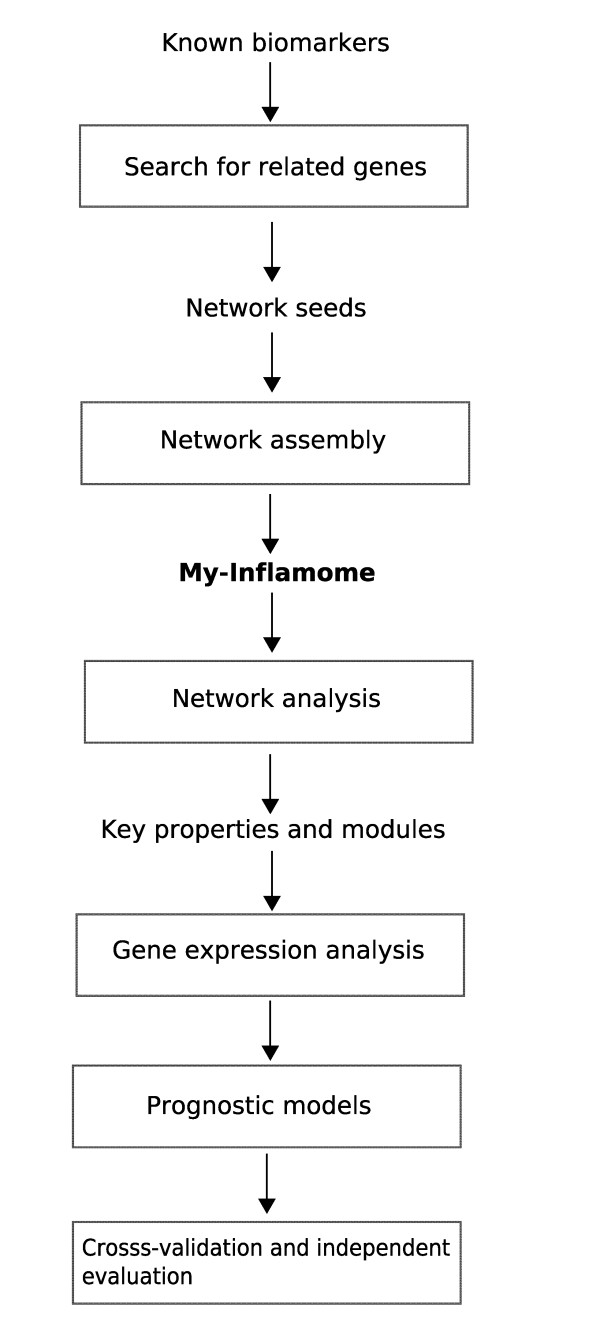
**Research framework and analytical pipeline implemented**.

Our investigation required the integration and analysis of multiples types of omic and clinical data, including external public information repositories and data generated at our laboratory. Figure 1 synthesizes the main phases and outputs of our research. First, using expert knowledge, we listed a set of well-known biomarkers in the context of inflammation and prognosis after MI. To reduce bias towards well-characterized biomarkers and to enable the discovery of new biological knowledge, this list of traditional biomarkers was expanded into a set of network *seeds*. This was accomplished by retrieving other functionally- and phenotypically-related genes from different public databases. These seeds were the product of a gene prioritization, which was established on the basis of their quantitative functional similarity to the traditional biomarkers. We then interrogated multiple protein-protein interaction (PPI) databases using the seeds as query inputs. This resulted in the extraction of their experimentally-validated PPIs in human, and the construction of a global network of PPIs in the MI and inflammation setting (*My-Inflamome*). This network was analyzed on the basis of different topological features to identify potential critical components and sub-networks.

The next phase aimed to answer the question of whether (and how) network-based information could be applied to aid in post-MI prognostic applications. More specifically, we investigated whether the genes encoding critical network components or involved in significant molecular modules could be applied to distinguish between post-MI clinical categories. To further demonstrate this translational research application, we built different classification systems based on microarray data from a small set of clinically meaningful genes. Models were evaluated using a standard cross-validation procedure. Furthermore, an experimental follow-up, using multiplex PCR, was conducted to assess opportunities for future research. These methods are described in more detail as follows.

### My-Inflamome seeds

The initial set of well-investigated inflammation biomarkers included those commonly applied in clinical practice, as well as others discussed in review articles in the post-MI context (Results) [2,3]. Genes functionally related to these biomarkers were searched in different public databases with the Endeavour system [20], which has previously been applied to different disease network investigations [16,21]. This is a gene prioritization task in which the training set is represented by the set of well-known biomarkers, and candidate genes originate from whole-genome searches. Functional similarity between candidate and training set genes is established using different types of omic information, such as annotation databases, gene sequences and public gene expression datasets. Thus, functional relatedness is estimated based on data source-specific similarity criteria. A global, multi-source similarity score is computed for each candidate gene across these resources, which is then used to rank candidate genes. The reader is referred to [21] for details about this gene search and prioritization procedure. We concentrated on candidate genes with global prioritization score, PS, equal to or lower than 0.01. The union of the sets of retrieved candidate genes and the well-known biomarkers represented the seeds of My-Inflamome.

### My-Inflamome construction

We obtained PPIs for the seeds using different public PPI databases: DIP [22], IntAct [23] and MINT [24] databases. These datasets have been shown to be complementary with high interactome quality and coverage [25]. We only retrieved PPIs validated in humans and focused on immediate (direct) seed-protein interactions. This aids in the reduction of the likelihood of retrieving PPIs not significantly related to the post-MI domain (false positives). The union of all retrieved PPIs defined My-Inflamome. We visualized and analyzed it with Cytoscape [26] and PolarMapper [27].

### My-Inflamome analysis

In My-Inflamome, proteins and their pair-wise interactions are graphically represented as network nodes and edges respectively. We analyzed fundamental network topological features at the node level: node degree and traffic. The former refers to the number of edges associated with a node. The latter, also referred to as "betweeness centrality", is the number of shortest paths that go through the node, and which link any two other nodes in the network.

A group of highly-interconnected nodes can be defined as a "module". A module may be identified through network clustering, which meets specific statistical analysis criteria as explained below. There are various statistical or topological features that can be used to characterize the networks, its modules and individual components. High traffic nodes indicate their "bottleneck" property, which has been correlated with different biologically-meaningful features, including putative disease biomarkers and drug targets [28-30]. In another study, we demonstrated the predictive potential of high traffic nodes in a global PPI network to detect patients with ventricular dysfunction [16].

Network modularity is another key topological property previously linked to disease biology and biomarker discovery [16,18,19,31]. A network module can be defined as a highly-interconnected sub-network, whose number of connections is larger than that expected from randomly pairing its proteins. We applied a "greedy" network clustering algorithm that maximizes a modularity score, *Q*, defined as: *Q = (numIME/numE) - (numIME/numE)_random_*, with *numIME *and *numE *representing the number of edges in a module and the total number of network edges respectively. We implemented network module identification and other topological analyzes with PolarMapper [27].

We characterized the resulting network modules on the basis of their association with Gene Ontology (GO) Biological Process (BP) and Cellular Component (CC) annotations. We also examined statistically detectable associations between microRNAs (miRNAs) and RNA targets in each module, and which are annotated in the miRBase database [32]. These associations were computed through (two-tailed) Fisher's exact tests and corrected to account for multiple-hypothesis testing using the Benjamini & Hochberg procedure. We concentrated on associations with (corrected) P < 0.01. We completed this functional characterization of modules with the Fatigo tool, under the Babelomics (v4.1) platform [33]. The *Statistica *software was used for standard statistical analysis [34].

### Prognostic biomarker discovery

To investigate the prognostic potential of critical nodes and modules, we examined the ability of their corresponding genes to distinguish between poor and good outcomes after MI (specified below). This was done on (blood-derived) gene expression data generated by microarray experiments at our laboratory (Gene Expression Omnibus, GEO, accession number: GSE11947) [35]. These data were obtained from circulating white blood cells, which play prominent roles in inflammation. Thus, we hypothesized that these cells better reflect inflammatory responses than secreted proteins. We first sought high traffic nodes and network modules showing strong differential expression capability, at the gene set level, across good and poor clinical outcome. The latter was estimated using the kipuMarkers approach [17,36]. Genes defining highest traffic nodes and predictive network modules were also used as inputs to classification models based on logistic regression (Ridge estimator value: 1.0E-8). Classification performance was summarized with the area under the receiving operating curve (AUC), and estimated with a leave-one-out cross-validation procedure (LOOCV). Classification models were implemented with the Weka system [37]. Inputs to classifiers were re-scaled so that their means and variances were equal to 0 and 1 respectively. Also note that we used the standardised outputs of the logistic regression classifiers to calculate the AUC values.

### Microarray gene expression data generation

We analyzed microarray gene expression profiles from 32 patients with good and poor post-MI clinical outcome (4 months after MI, 16 patients/category). Good outcome was defined as the preservation of left ventricular (LV) systolic function and high ejection fraction (EF) after MI (EF > 40%, median 63%, range 45-73%). A patient exhibited poor outcome when presenting impaired LV function and low EF (EF ≤ 40%, median 35%, range 20-40%). Clinical characteristics of patients selected, microarray data generation and pre-processing procedures are described in [18].

### Experimental verification

We also performed a follow-up experimental replication using multiplex TaqMan PCR. Out of the 32 RNA samples extracted from the patients used in the microarray experiments, 11 samples were available for quantitative PCR determination. Thus, we measured the gene expression of key putative biomarkers using this sub-set of patients (11 patients, 7 with poor clinical outcome) as a first validation step.

### Multiplex TaqMan assay

Total RNA was extracted from 2.5 mL of whole blood collected the day of MI by the PAXgeneTM technology and 1 μg of total RNA was reverse transcribed using SuperScriptTM II Reverse Transcriptase (Invitrogen, Merelbeke, Belgium) according to the manufacturer's protocol. Expression levels of TRAF2 (TNF receptor-associated factor 2), UBC (ubiquitin C) and SHKBP1 (SH3KBP1 binding protein 1) were assessed by quantitative PCR, in a multiplex TaqMan assay. SF3A1 was chosen as housekeeping gene for normalization. PCR was performed in a BioRad CFX96™ apparatus using the following primers and probes: TRAF2 forward primer GGCTTCTCCAAGACCCTCCTG, TRAF2 reverse primer TTCGTGGCAGCTCTCGTATTCT, TRAF2 probe TexasRed-ACTGTGCTGCCTGTGTTCACGAG-BHQ2, UBC forward primer AGCGAGCGTCCTGATCCTTC, UBC reverse primer CACCCGGCGCGTCCTTATAT, UBC probe FAM-AGTAGTCCCTTCTCGGCGATTCTG-BHQ1, SHKBP1 forward primer GACGGAGCAAGAGCTGATGGA, SHKBP1 reverse primer GCTGCGTTGTTCAAAAGGAAGTTT, SHKBP1 probe HEX-CCAGGAACTGGTGCGGAGTGG-BHQ2, SF3A1 forward primer AAGGGTCCAGTGTCCATCAAAGT, SF3A1 reverse primer GCCATGTTGTAGTAAGCCAGTGAG, SF3A1 probe Cy5-ACCAGGTCTCTGTCATTAAGGTGAAG-BHQ3. The multiplex reaction was performed on 4 μL of cDNA (previously diluted 10×) in a 20 μL reaction mix containing 1× AmpliTaq Gold buffer (Applied Biosystems, Halle, Belgium), 5 mM of MgC_l2 _(Applied Biosystems), 500 μM of each dNTPs (Invitrogen), 4 U of AmpliTaq Gold (Applied Biosystems), 400 nM of each TRAF2 primers and probe, 300 nM of each SHKBP1 primers and probe, 1 μM of each UBC primers, 500 nM of UBC probe and 200 nM of each SF3A1 primers and probe. The PCR reaction was composed of 1 cycle at 95°C for 8 min, followed by 60 cycles at 95°C for 30 s, 63.5°C for 30 s and 72°C for 30 s. Primer concentrations and annealing temperature were optimized to allow for multiplex detection. Regression was used as Ct determination mode for data analysis. Expression of TRAF2, UBC and SHKBP1 genes was calculated as: gene expression = 2^(Ct SF3A1 - Ct gene)^.

In order to select the housekeeping gene for this experiment, we measured the expression of 12 different known housekeeping genes: ACTB, GAPDH, UBC, B2M, YWHAZ, SF3A1, 18S rRNA, CYC1, EIF4A2, SDHA, TOP1 and ATP5B, using the geNorm™ Housekeeping Gene Selection Kit (PrimerDesign). Results indicated that the expression of SF3A1 was the most stable between samples. We determined the amplification efficiency of each primer pair during the set up of this multiplex TaqMan qPCR essay: efficiencies varied from 93.1% to 102.8%, depending on the primer pair studied. This indicated high amplification efficiency for each gene. Moreover, we compared the expression of each gene in the multiplex reaction to its expression in a singleplex reaction. Ct values obtained for each gene were identical between multiplex and singleplex PCR, indicating that the multiplex reaction was not rate limiting.

## Results

### My-Inflamome: A novel resource for inflammation and post-MI research

**Figure 2:**
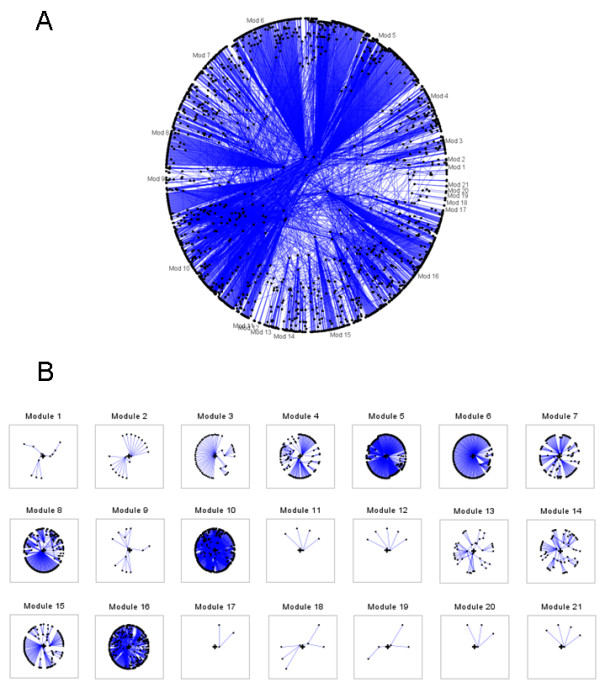
**My-Inflamome network**. A. Global view. B. Modular view.

**Figure 3:**
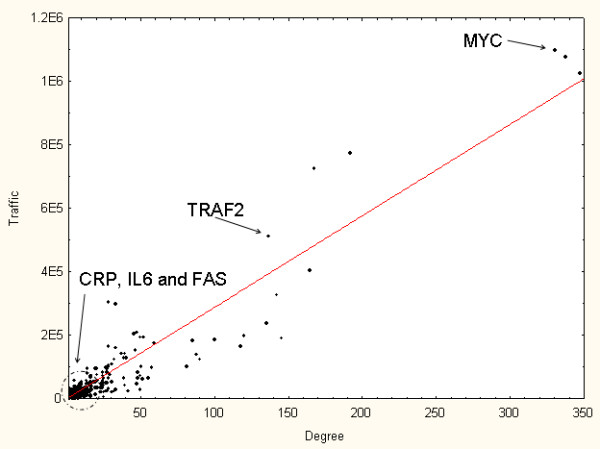
**Correlation between node degree and traffic in My-Inflamome**.

My-Inflamome seed set consisted of 415 proteins, including 28 known biomarkers. Examples of seed proteins are: TNF, CRP, FAS and TNFRSF10A. My-Inflamome was composed of 2595 proteins and 6181 interactions in total. The single largest, interconnected network component (island) included 2532 nodes and 6131 interactions (mean number of interactions per node: 4.25). We focused on this network for subsequent analyses (Additional file 1). A global view of My-Inflamome is displayed in Figure 2. Figure 3 depicts the correlation between node degree and traffic in My-Inflamome. Examples of known and candidate biomarkers are highlighted on the plot. Because of the observed relatively strong correlation (Spearman correlation, ρ = 0.93, P = 1E-4) and the previously demonstrated functional importance of high traffic nodes, we concentrated on top high-traffic proteins in subsequent analyses (Table 1). We note that none of the top-10 high-traffic nodes were included in the list of known inflammation biomarkers in post-MI. On the basis of their traffic values, standard inflammation biomarkers CRP, IL6 and TNF ranked 227^th^, 478^th ^and 607^th ^respectively. This further indicates that our approach is not biased toward traditional biomarkers.

**Table 1:** Top-10 high-traffic proteins in My-Inflamome.

Protein	Degree	Traffic
MYC	330	1.10E+06

IKBKE	337	1.08E+06

TRAF6	347	1.03E+06

TP53	192	7.77E+05

EGFR	167	7.26E+05

TRAF2	136	5.13E+05

MAP3K3	164	4.07E+05

IKBKG	142	3.28E+05

GRB2	28	3.08E+05

UBC	33	2.99E+05

### My-Inflamome is biologically meaningful

We detected 21 highly-interconnected modules with diverse sizes and numbers of interactions in My-Inflamome (Table 2). The largest modules are strongly implicated in post-translational protein modifications and signal transduction processes (e.g., Modules 5, 10 and 16). Different modules are directly relevant to cell death and development processes (e.g., Modules 4, 8 and 14). Others appear specialized in immune responses (e.g., Module 9). Four modules were not statistically associated with GO BP terms (Modules 11, 12, 17 and 21). Module 1 is specialized in blood coagulation and wound healing processes. My-Inflamome modules operate across different cellular regions, including the nucleus, cytoplasm and the extra-cellular space. However, 7 modules were not significantly associated with specific cellular compartments.

**Table 2:** Topological description of My-Inflamome modules.

Module	NP	IntraMI	InterMI	TotInt	MTraffic
1	11	10	8	18	38

2	19	18	21	39	36

3	52	52	41	93	102

4	132	153	122	275	262

5	437	587	391	978	872

6	252	268	212	480	502

7	127	148	105	253	252

8	265	317	183	500	528

9	13	15	7	22	24

10	507	1589	610	2199	1012

11	5	4	1	5	8

12	6	5	7	12	10

13	39	49	11	60	82.16

14	65	84	41	125	128

15	118	131	59	190	234

16	460	863	307	1170	918

17	3	2	2	4	4

18	7	6	2	8	12

19	5	4	2	6	8

20	4	3	1	4	6
21	5	4	1	5	8

NP: Number of proteins, IntraMI: Number of intra-module interactions, InterMI: Number of inter-module interactions, TotInt: Total number of interactions, MTraffic: Median traffic.

**Figure 4:**
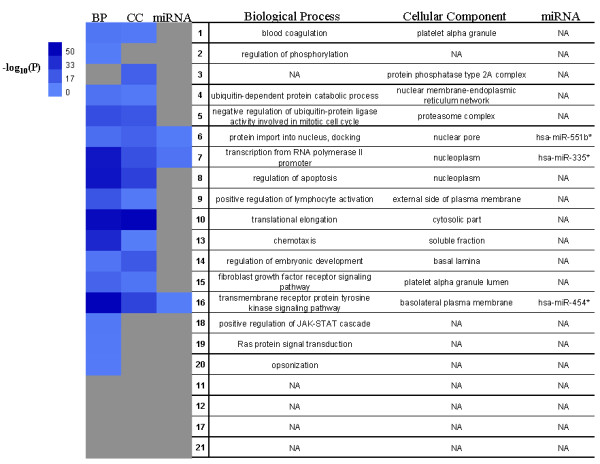
**Functional characterization of My-Inflamome modules**. Most statistically detectable associations with GO BP, CC and microRNAs. In heat map: P is the probability associated with the functional category observed in each module, colors reflect log-transformed values of P. BP: biological process, MF: molecular function, CC: cellular component. NA: No statistically detectable association. Modules are numbered as in Figure 2. The darker the color, the more significant the statistical association.

Because of the therapeutic and prognostic potential of these modules, we searched for major interactions between miRNAs and targets found in each module. We found statistically detectable evidence that Modules 6, 7 and 16 may be regulated by different miRNAs. For example, hsa-miR-335*, recently implicated in breast tumor suppression [38], is substantially linked to targets in Module 7, which in turn are significantly enriched in genes with transcriptional regulatory roles. Figure 4 summarizes this functional quantitative characterization of My-Inflamome modules on the basis of the most significant associations observed. Details of module composition and functional descriptions are available in Additional file 2.

### My-Inflamome guides the discovery of candidate prognostic biomarkers

To assess the predictive potential of these results, first we established the capacity of genes encoding high-traffic proteins and of modules to differentiate prognostic categories (good vs. poor prognosis). Among the top high-traffic proteins, IKBKE (inhibitor of kappa light polypeptide gene enhancer in B-cells, kinase epsilon), TRAF2 (TNF receptor-associated factor 2) and UBC (ubiquitin C) were found statistically differentially expressed at the (nominal) P = 0.02 level (Mann-Whitney U Test). The modules showing the highest differential (mean) expressions were Module 17 (corrected P = 0.02) and Module 11 (P = 0.06). Note that these modules are not statistically linked to specific biological processes, and only Module 11 appears circumscribed to a specific cellular region (Collagen type VI). Within Module 11, SHKBP1 (SH3KBP1 binding protein 1) is differentially expressed (Mann-Whitney U Test, nominal P = 0.006).

Motivated by these findings, we evaluated these genes and modules as inputs to (supervised) computational prognostic models. Different combinations of genes and modules were used as inputs to logistic regression models (Table 3). The highest classification performance (AUC = 0.84) was obtained when using the individual expression values measured in M11 and M17 (6 genes with available microarray data: CD5, PIK3C3, FBXL2, MIA3, SHKBP1 and ZC3H7A). This was followed by a model in which the mean expression values of M11 and M17 were applied as inputs (AUC = 0.8). The combination of module-derived and high-traffic genes also offered promising results (TRAF2, SHKBP1 and UBC, AUC = 0.83).

**Table 3:** Summary of classification performance of models defined by My-Inflamome information.

Model number*	Inputs	AUC
1	Mean expression values of M11 and M17	0.80

2	Individual expression values of M11 and M17	0.84

3	TRAF2, SHKBP1, UBC	0.83

*Logistic regression classifiers.

### Replication analyses

To verify the validity of these candidate biomarkers, we replicated microarray experiments using an independent platform, quantitative PCR (Methods). In this first follow-up evaluation, we decided to focus on the prognostic signature defined by TRAF2, SHKBP1 and UBC. As a first classification model verification strategy, we tested the model derived from the microarray data on the multiplex PCR data (11 samples). The classification performance on this dataset was comparable to that obtained above (AUC = 0.79). In a second verification strategy, we re-built the classification model on the 11 PCR data samples and estimated its performance using LOOCV. This evaluation also reported classification capability comparable to previous performance using this signature (AUC = 0.77). Although this does not qualify as an independent evaluation of these potential novel biomarkers, these results at least suggest that it is viable to obtain adequate predictive concordance between prognostic models when tested on different data generated by independent expression measurement platforms.

## Discussion

### Significance

Although inflammation biomarkers are useful to support medical decision-making in the MI setting, the complexity and roles of the global interactions of these proteins have not been adequately elucidated. This is partly explained by the lack of systems-based approaches to bridge clinical (i.e., disease phenotypes) and molecular information (i.e., different types of omic information). The rationale for applying integrative, network-driven approaches is reinforced by the knowledge that the interactive activity of inflammation proteins may be functionally critical to multiple biological processes, which cannot be pinpointed by traditional hypothesis-driven research. Moreover, such an inflammation interactome is spatially distributed in the cell and subject to different regulatory control mechanisms acting in a combinatorial fashion. With an approach solely relying on traditional hypothesis-driven research or single-source molecular data, it would be difficult to accurately establish relevant connections between biomarkers and their prognostic value, while at the same time providing adequate mechanistic visualizations. In this paper, we laid out a foundation to address these challenges and discover potentially novel translational research applications.

The inflammation-related proteins TRAF2, SHKBP1 and UBCs were here associated with clinical outcome after MI based on a multi-source, functional characterization of network-derived properties. To accomplish this, we assembled a new compendium of interacting proteins that are interrelated, and not limited to known inflammation biomarkers in post-MI. My-Inflamome represents by itself a contribution to enable future independent investigations.

Despite their established use, standard inflammation markers are poor predictors of clinical outcome after MI. Prior to this investigation we did not have evidence to suggest that known biomarkers would appear as top high-traffic nodes. Our research indicates that indeed standard biomarkers do not necessarily act as network bottlenecks in the inflammation network. This confirms the known little predictive power of widely-applied inflammation biomarkers as shown by others and our previous research. Thus, our results offer a possible explanation for the lack of predictive capability of traditional markers: They have limited roles as central mediators or coordinators of inflammation responses in MI.

Among these putative biomarkers, SHKBP1 (SH3KBP1 binding protein 1) has not been widely characterized, though SH3KBP1 (SH3-domain kinase binding protein 1) is known to be implicated in apoptosis. Moreover, SHKBP1 has not been specifically linked to cardiovascular disease prior to our study. We found this new relationship by extracting SHKBP1 from a My-Inflamome module. Although this module was not statistically associated with specific biological processes or even specific cellular localizations, the combined expression values of the genes encoding this module's members showed relatively powerful prognostic capacity. SHKBP1 is over-expressed in patients with poor prognosis, and can actually offer relatively good classification performance when used as a single biomarker (AUC = 0.75, microarray data). Although not a necessary condition for effective integrated (multi-gene) classification, relatively strong differential expression of individual inflammation biomarkers may add to the potential clinical value of the biomarkers. Inflammation is a natural reaction to myocardial injury and the duration of these events largely determine heart remodelling. Thus, future research could offer more detailed quantitative relationships between individual levels of inflammation genes and their influence on cardiac damage or remodelling.

Inflammation can cause plaque rupture in the coronary arteries, which leads to MI. However, this type of inflammation is highly localized in the heart. Therefore, we cannot confer putative causative roles to our proposed circulating biomarkers.

Good classification performance was observed when replicating results with PCR data obtained from the same cohort of patients used in the model derivation phase. These results encourage additional investigations on independent, larger cohorts.

It is also crucial to indicate that standard clinical biomarkers, N-terminal pro-brain natriuretic peptide (NT-pro-BNP) and troponin T, have already been analyzed on the same (model derivation) cohort here investigated [18]. Prognostic models based on these markers reported lower performance (LOOCV, AUC < 0.7) than those obtained here with TRAF2, SHKBP1 and UBC.

An examination of the literature and annotated disease databases indicates that TRAF2, SHKBP1 and UBC have not been widely investigated in the context of cardiovascular disease, and that no quantitative links have been made to clinical response after MI. Only UBC has been previously linked to coronary artery disease severity [39]. Also we detected promising associations between miRNAs and My-Inflamome that could guide the design of new prognostic or therapeutic interventions based on "driving" regulatory modulation strategies. Considering that we did not incorporate expression information of miRNAs, the establishment of such statistically detectable associations underscores the predictive potential of our approach.

Overall, these observations underline the value of our approach to uncover potentially clinically relevant associations, which can be both novel and unbiased.

### Limitations and future research

The level of certainty of our quantitative associations is constrained by the size of the datasets used in the model derivation and verification analyses of prognostic models. Nevertheless, we expect to expand our performance estimations and comparisons with alternative models as more data become available. Moreover, in this study we showed the feasibility of producing concordant results when using different data generation platforms.

It may be argued that My-Inflamome does not include all components with relevant involvement in inflammation-mediated responses to cardiac injury and remodeling. We acknowledge that the rate of false negatives may be reduced by implementing a more inclusive network inference process, e.g., by expanding the seed set. However, at this stage we emphasized quality, rather than coverage, to control the level of potentially spurious or false positive associations.

The clinical acceptance of new biomarkers firstly depends on the successful development of larger, independent evaluations. Moreover, future assessments should involve additional comparisons with standard post-MI biomarkers, including those not directly connected to inflammatory responses (e.g., NT-pro-BNP) [3]. Future research should incorporate measurements of standard clinical biomarkers, such as CRP or TNF-α [2]. To enhance the scientific exploitation of My-Inflamome and add value to our findings, we also plan to facilitate their user-friendly access and analysis via a Web-based interface. Researchers can currently have full access to My-Inflamome in a text-formatted file format for non-commercial application (Additional file 1).

## Conclusion

In this research, we demonstrated how an integrative, network-driven strategy can improve systems-level mechanistic understanding of inflammation in MI. We also showed the translation of network-derived information into new knowledge with potential applications to assess prognostic outcome after MI. In particular, we reported new prognostic associations involving three genes: TRAF2, SHKBP1 and UBC. Potential clinical implications of our results, including the proposed biomarkers, will require independent studies.

## List of abbreviations used

AUC: Area under the receiver operating characteristic curve; BP: GO biology process hierarchy; GO: Gene Ontology; HF: Heart failure; LOOCV: Leave-one-out cross-validation; MI: Myocardial infarction (MI).

## Competing interests

The authors declare that they have no competing interests.

## Authors' contributions

FA and DW conceived the study. FA designed the discovery strategy and implemented computational analyses. LZ implemented computational analyses. SR performed expression measurement experiments with multiplex PCR. DW and YD provided biomedical feedback. FA wrote the manuscript assisted by the other authors. All the authors read and approved the manuscript.

## Pre-publication history

The pre-publication history for this paper can be accessed here:

http://www.biomedcentral.com/1755-8794/4/59/prepub

## Supplementary Material

Additional file 1 **My-Inflamome network data**. Click here for file

Additional file 2 **Functional modular characterization of My-Inflamome**. Click here for file
